# Identification of phytochemical, antioxidant, anticancer and antimicrobial potential of *Calotropis procera* leaf aqueous extract

**DOI:** 10.1038/s41598-023-42086-1

**Published:** 2023-09-07

**Authors:** Armin Ahmad Nejhad, Behrooz Alizadeh Behbahani, Mohammad Hojjati, Alireza Vasiee, Mohammad Amin Mehrnia

**Affiliations:** 1 Department of Food Science and Technology, Faculty of Animal Science and Food Technology, Agricultural Sciences and Natural Resources University of Khuzestan, Mollasani, Iran; 2 Department of Food Science and Technology, Faculty of Agriculture, Ferdowsi University of Mashhad, Mashhad, Iran

**Keywords:** Biochemistry, Microbiology, Pathogenesis

## Abstract

Since the dawn of civilization, people have turned to plants as a safe and efficient form of treatment for a variety of diseases. It has long been known that *Calotropis procera* has the potential to treat a number of diseases. In this study, the *C. procera* leaf aqueous extract was obtained using the maceration method, and *p-*coumaric was found to be the main compound. The extract was rich in phenols (174.82 mg gallic acid equivalent/g) and flavonoids (1781.7 µg quercetin equivalent/g). The extract had high antioxidant properties, as indicated by the IC_50_ values obtained for 2,2-diphenyl-1-picrylhydrazyl (DPPH) (366.33 μg/mL) and 2,2′-azino-bis (3-ethylbenzothiazoline-6-sulfonic acid (ABTS) (169.04 μg/mL), as well as the ferric ions reducing antioxidant power (FRAP) (1.67 μg ascorbic acid equivalent/g of the extract). The cytotoxicity of the extract was evaluated against the survival of HT 29 cells, and the IC_50_ was found to be 236.87 μg/mL. The most resistant and sensitive strains to the extract were *Escherichia coli* and *Staphylococcus aureus*, respectively. The morphological changes of these strains were demonstrated through scanning electron microscopy and confocal laser scanning microscopy. The *C. procera* extract could be therefore used as an antioxidant, antimicrobial, and anticancer agent.

## Introduction

The public has become increasingly concerned about the toxic effects of synthetic and chemical additives, which are used for their antimicrobial, antioxidant, and flavoring properties. In response to this concern, researchers have been exploring natural alternatives to these additives. One promising option is the use of natural plant extracts, which contain a wide range of flavoring, antimicrobial, antioxidant, and anticancer substances.

*Calotropis procera*, also known as Stabregh in Iran, is a plant from the *Apocynaceae* family^1^. It is also known by various other names such as Aiton, Sodom apple, Milkweed, Calotrope, Giant milkweed, Indian milkweed, Wild cotton, Rubber tree, Usha, Aak, and Madar^2–7^. This plant can be found in tropical areas and southern coasts of Iran, North Africa, Middle East Asia, and Southeast Asia on coastal sand dunes^4,7^. All parts of the plant (root, stem, leaf, and flower) have various uses in industries such as food processing, pharmaceuticals, fuel production, textile, paper production, rubber production, plant treatment, and synthesis of nanoparticles^6,8^. It is also widely used in traditional medicine systems in the treatment of skin diseases, leprosy, asthma, rheumatism, malaria, and traditional indigestion^5,9^. The plant contains compounds such as flavonoids, phenolics, steroids, cardenolides, tannins, terpenoids, saponins, alkaloids, and terpenoids, which have bactericidal, fungicidal, insecticidal, anti-cell toxicity, anti-inflammatory, and antioxidant properties^4^. The antioxidant, antibacterial, antifungal, anti-inflammatory, anticancer, antidiabetic, and antimalarial properties of *C. procera* and its extracts have comprehensively been reviewed by Dogara^10^. In West Africa *C. procera* extract is used to coagulate milk and produce cheese. Moreover, one of the significant uses of *C. procera* latex is its effectiveness in treating hair loss and dental issues^5^.

Phenolic compounds are the most important antioxidant compounds of plant extracts. They inactivate hydroxide and peroxide radicals through electron donation, which can cause cytotoxicity and cancer when they react with human body cells. In addition, plant extracts contain antimicrobial compounds that are a suitable alternative to antibiotics, which have developed resistance to a wide range of them in recent years^11^.

The characteristics of the *C. procera* plant have not been fully investigated so far. Therefore, this study aims to investigate the plant’s characteristics due to its importance in the food industry. This work differs significantly from other existing research articles as it provides a comprehensive analysis of the full biological characteristics of the aqueous extract of the native *C. procera* plant. This includes its chemical constituents, antioxidant activity, anti-cancer effect, antimicrobial effect, and mechanism of action of its antimicrobial activity, all in one place. In this study, the chemical composition of *C. procera* extract was analyzed using high performance liquid chromatography (HPLC) and Fourier transform infrared spectroscopy (FTIR) tests. The total phenol content (TPC), total flavonoid content (TFC), total beta-carotene content, antioxidant properties, and antimicrobial effect of the extract were also measured. The cytotoxicity factor of the extract was measured to evaluate its anticancer property. Finally, the effect of the extract on the structure of *Escherichia coli* and *Staphylococcus aureus* was investigated using scanning electron microscopy (SEM) and confocal laser scanning microscope (CLSM). This study aims to explore the potential applications of *C. procera* as a novel platform for the treatment of various diseases.

## Material and methods

### Chemicals, microorganisms, and culture media

The DPPH (2,2-diphenyl-1-picrylhydrazyl), Folin-Ciocalteu reagent, gallic acid, ABTS (2,2′-azino-bis(3-ethylbenzothiazoline-6-sulfonic acid)), potassium persulfate, and dimethyl sulfoxide (DMSO) were purchased from Merck Co. (Darmstadt, Germany). The strains of *Listeria monocytogenes*, *Bacillus cereus*, *Salmonella typhimurium*, *S. aureus*, *E. coli*, *Shigella dysentery*, and *Staphylococcus epidermidis* were obtained from National Center of Biological and Genetic Resources of Iran. Mueller Hinton broth (MHB), Mueller Hinton agar (MHA), nutrient agar (NA), and tryptic soy broth (TSB) were purchased from Difco Laboratories (Detroit, MI, USA).

### Inoculum development

The preparation of stock culture of pathogenic bacteria was done using MHB and NA culture media. *L. monocytogenes* strains were inoculated in TSB culture, while other strains were grown on agar slants from their own culture medium for 24 h at 37 °C. After incubation, the cultures were kept at 4 °C^12^.

### Preparation of *C. procera* leaf extract

The *C. procera* plant was collected from its habitat in the south of Iran and was identified by Dr. Kazem Negaresh at the herbarium center of Agricultural Sciences and Natural Resources University of Khuzestan (herbarium code, KHAU319). The experimental field study was conducted in compliance with relevant institutional guidelines and regulations. The initial preparation of the plant leaf involved washing, drying (under suitable temperature and humidity conditions in the shade), and grinding using a laboratory mill. The maceration method was then conducted by soaking the plant in water (1:10 ratio; w/v) for 24 h at ambient temperature with stirring. The extract was separated from the plant using filter paper, and centrifugation and evaporation were performed with a rotary. The resulting extract was kept at 4 °C for further analysis^11^.

### Phenolic profile by HPLC

According to a previous study by Yeganegi et al.^13^, the extract's polyphenol content was determined by HPLC (Knauer, Germany) using a C18 column (4.6 mm ID × 150 mm (5 µm) and a UV detector (at 350 nm) as follows. Elution profile: A = 0.15% phosphoric acid in H_2_O–MeOH 77:23 (v/v, pH = 2); B = MeOH. Isocratic: 0–3.6 min 100% A; gradient: 3.6 min 100% A-linear- 24.0 min; 80.5% A-isocratic-30 min linear- 60 min; 51.8% A-linear-67.2 min; 100% B; flow rate: 1.0 ml/min. The injected volume was 20 µL. The relative retention indices were computed due to the low reproducibility of the retention times in HPLC chromatograms. By comparing the relative retention indices of the peak with those that had already been published and by co-injecting naringenin, quercetin, kaempferol, apigenin, and rutin, the peak identity was confirmed. From the HPLC peak areas, the percentage composition of the extracts was determined.

### Functional group analysis by FTIR

The FTIR spectrum was collected for sample at room temperature in the 400–4000 cm^-1^ range by a FTIR spectrophotometer (Perkin Elmer, USA)^14^.

### Measurement of TPC and TFC

The TPC and TFC of the extract were determined using the method described by Behbahani et al.^11^. A mixture containing 20 µL of extract (concentration: 10 g/L), 2 mL of distilled water, and 100 µL of Folin-Ciocalteu reagent was prepared. After 3 min, 300 µL of sodium bicarbonate solution was added to the mixture, which was then stirred for 2 h. The absorbance of the sample was recorded at 765 nm in a spectrophotometer (Sigma3, 30k). A standard curve was drawn using gallic acid (0–500 mg/L). The final TPC value was reported in terms of mg gallic acid (GAE)/g of extract^11^.

To measure the TFC, 1 mL of the crude extract concentration was mixed with 1 mL of 2% methanolic aluminum chloride, kept for 15 min at room temperature in the dark, and the absorbance values of the samples were measured at 430 nm. The quercetin was used to draw the standard curve, and TFC value was reported as μg quercetin (QE) equivalence/g of dry weight of extract^11^.

### Beta-carotene content

The beta-carotene content of the sample was measured using HPLC (Knauer, Germany, a C18 column (4.6 mm ID × 150 mm (5 μm)), with a UV detector (at 350 nm))^15^.

### Antioxidant activity evaluation

#### DPPH free radical scavenging assay

The procedure described by Yeganegi et al. (2018) was used to determine the antioxidant activity of the extract. A methanolic solution of the extract was prepared to reach a concentration of 1 mg/mL, and then diluted to a range of 10–500 μg/mL. Solutions of 1 mL methanol containing 0.2 mM DPPH were made, and 1 mL of each extract concentration was added. The samples were placed in the dark for 30 min at 24 °C, and the absorbance of the extract and blank samples (all components of the reaction without extract) was measured at 517 nm. The antioxidant activity was calculated using the following formula:

$${\text{I}}\% = \left( {A_{{{\text{blank}}}} {-}A_{{{\text{sample}}}} {/}A_{{{\text{blank}}}} } \right) \times {1}00$$

where A _sample and_ A _blank_ indicate the absorption of extract sample and blank sample, respectively. The antioxidant activity of the extract was compared to that of natural and synthetic antioxidants, vitamin C and tert-Butylhydroquinone (TBHQ), using the IC_50_ value^13^. The IC_50_ value represents the concentration of the sample capable of inhibiting 50% of free radicals and is calculated using the slope equation of the radical scavenging activity (RSA) curve.

#### ABTS free radical cation scavenging assay

The extract’s ability to inhibit ABTS radicals was measured using the protocol of Labiad et al. (2017). Vitamin C and TBHQ were used as controls. A free radical solution (7 mM ABTS, 2.4 mM potassium persulphate) was prepared and left in the dark for 14 h at 24 °C. Extract samples (200 µL; 10–500 μg/mL) were added to the free radical solution (2 mL) and mixed completely. After 30 min, the absorbance of the samples was read at 734 nm and the ABTS free radical scavenging activity was reported in terms of IC_50_^16^.

#### Ferric reducing antioxidant power (FRAP) assay

The FRAP assay was used to measure the extract’s antioxidant power. A solution containing 0.2 M phosphate buffer (2.5 mL; pH 6.6) and potassium ferricyanide (2.5 mL; 1% w/v) was prepared and charged with the extract and incubated at 50 °C for 20 min. The reaction was stopped by adding trichloroacetic acid (2.5 mL; 10% w/v) and centrifugation (1000×*g* for 10 min) was then conducted. A mixture consisting of 2.5 mL of supernatant, 2.5 mL of deionized water, and 0.5 mL of 0.1% chloride was prepared and after 30 min, the absorbance of the sample was read at 700 nm. The FRAP of the extract was reported in terms of ascorbic acid equivalent (mg AAE/g of extract). Vitamin C and TBHQ were used as positive controls^16^.

#### Cytotoxicity studies

The MTT (3-(4,5-dimethylthiazol-2-yl)-2,5-diphenyltetrazolium bromide) assay was used to measure the cytotoxicity of the extract against the HT29 cell line (IBRC cell number C10097, National Center for Genetic and Biological Resources of Iran). The cells were cultivated in DMEM medium with 10% fetal bovine serum and penicillin/streptomycin followed by incubation at 37 °C, 95% humidity, and 5% carbon dioxide. Cells (100,000 cells) were added to wells and different doses of extract (, 12.5, 25, 50, 100, 200, 400, and 800 μg/mL), the DMEM culture medium, and 200 µL of fetal bovine serum were added to each well. The cell proliferation was measured using the MTT method after 24 h of incubation as follows: 30 µL of MTT solution with a concentration of 5 mg/mL was added to each of the wells, and the plates were incubated for 3 h in a carbon dioxide incubator, and the absorbance of the medium was read at 570 nm using an ELISA reader (ELX 808, Bio Tek Instruments, USA). The cell viability curve was drawn using control cells^17^.

### Antimicrobial activity evaluation

#### Disc diffusion agar (DDA) and well diffusion agar (WDA) methods

In the DDA method, different concentrations of the extract were prepared and sterilized. The discs were immersed in the extract and then placed in the culture medium inoculated with target bacteria. The medium was incubated at 37 °C for 24 h. The inhibition zone (IZ) around the discs was measured as an indicator of antimicrobial activity. Ciprofloxacin antibiotic was used as a positive control^11^.

In the WDA procedure, the microbial suspension was inoculated in the MHA culture medium and distributed uniformly. Wells with a diameter of 6 mm were created on the medium and 20 µL of each extract concentration was added to the wells. The medium was incubated at 37 °C for 24 h and the IZ was reported as an indicator of antimicrobial activity of the extract^11^.

#### Minimum inhibitory/bactericidal concentration (MIC/MBC)

The MIC and MBC were measured using the microdilution method according to the National Committee for Clinical Laboratory Standards (NCCLS). The method involved preparing a primary culture of pathogenic bacteria at a concentration of 1.5 × 10^8^ CFU/mL and diluting the extract containing DMSO (up to 1 mg/mL) with MHB. Bacteria were added to each well of a 96-well plate and 125 µL of the extract was poured into each well. The plate was incubated for 24 h at 37 °C. Triphenyltetrazolium chloride (25 µL; 5 mg/mL) was added to each well and the formation of a dark red color, indicating the growth of microorganisms, was checked. The concentration of the extract in which no color change was observed was considered as the MIC. The contents of each well (100 μL) where no color change was observed were cultured on MHA and incubated at 37 °C for 24 h. The minimum dilution that prevented growth was considered as MBC^11^.

#### Evaluation of the structure of *E. coli* and *S. aureus*

To evaluate the effect of the *C. procera* extract on the structure of *E. coli* and *S. aureus*, SEM (LEO 1450 VP model, Germany) images were prepared. After strain cultivation and incubation, the sample was centrifuged and washed using 0.1 M sodium phosphate buffer (pH 7) and polycarbonate filter to remove impurities. The strain was fixed with glutaraldehyde and was placed at 4 °C for 2 h. Distilled water and ethanol were used for final washing, and the sample was dried in vacuum. Finally, a gold layer was placed on the sample and SEM images were prepared^18^.

The effect of *C. procera* extract on the biofilm formation of *E. coli* and *S. aureus* was evaluated using a CLSM based on the method of Bandara et al. (2013). Presterilized flat bottom six-well plates (Iwaki) and plastic coverslips (Thermanox plastic coverslips; Nulge Nunc International, Rochester, NY, USA) were used^19^.

### Statistical analysis

Statistical analysis was performed using one-way analysis of variance (ANOVA) with SPSS software (version 17, SPSS Inc., Chicago, IL). Means were further classified using Tukey as a post test. P values of 5% were considered significant.

## Results

### Phenolic profile of the extract

Table 1 presents the results related to the components of the *C. procera* extract. The main substances identified were catechin, rutin, p-coumaric acid, caffeic acid, luteolin, and kaempferol. The highest concentration was found for p-coumaric acid (396.2 μg/g DW) while the lowest concentration was accounted for rutin (142.4 μg/g DW).

**Table 1:** The main composition of *C. procera* crude extract obtained by HPLC.

Phenolic compounds	Concentration (µg/g DW)
Catechin	275.2
Rutin	142.4
*P*-Cumaric acid	396.2
Caffeic acid	308.2
Luteolin	295.6
Kaempferol	157.9

### FTIR spectrum of the extract

**Figure 1:**
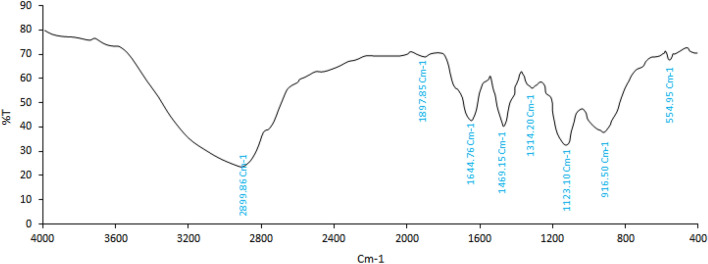
FTIR spectrum of *C. procera* extract.

The chemical bonds in the extract compounds were analyzed using FTIR (Fig. 1). The stretching vibrations of CH_2_ groups in alkanes were identified at a peak of 541 cm^−1^. A peak in the wavenumber range of 1000–1332 cm^−1^ indicates the presence of C–O bonds in alcohols, carboxylic acids, esters, and ethers. The signal at 1476 cm^−1^ is attributed to the C=C stretching vibrations of an aromatic ring^8,20^. The peak at 1685 cm^−1^ is associated with the C=O vibrations of aldehyde groups. The C–H stretching vibrations, that is often related to alkaline compounds in the extract, were found at 2955 cm^−1^^18^. Peaks in the range of 3000–3500 cm^−1^ are related to the stretching vibrations of hydroxyl (O–H) groups, which may be associated with alcohol groups or carboxylic acids^17^.

### TPC, TFC, and beta-carotene content

**Figure 2:**
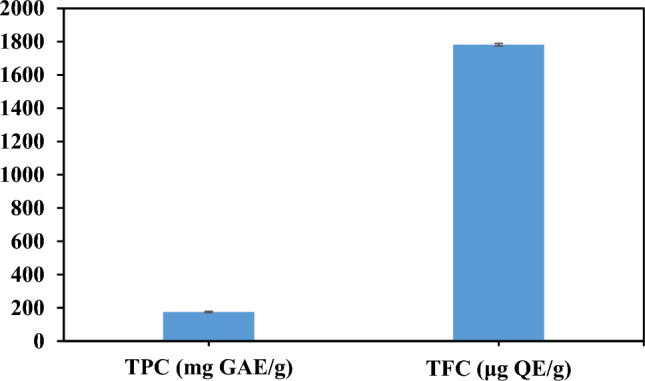
Total phenolic content (TPC) and total flavonoid content (TFC) of *C. procera* extract.

The results of TPC and TFC of the extract are provided in Fig. 2. The TPC of the extract was found to be 174.82 ± 3.60 mg GAE/g of DW. Moreover, the TFC value for the *C. procera* extract was 1781.7 ± 7.64 µg QE/g of DW. Also, the amount of beta-carotene of the extract was calculated to be 2.26 ± 0.05 mg/100 g.

### Antioxidant potential

**Figure 3:**
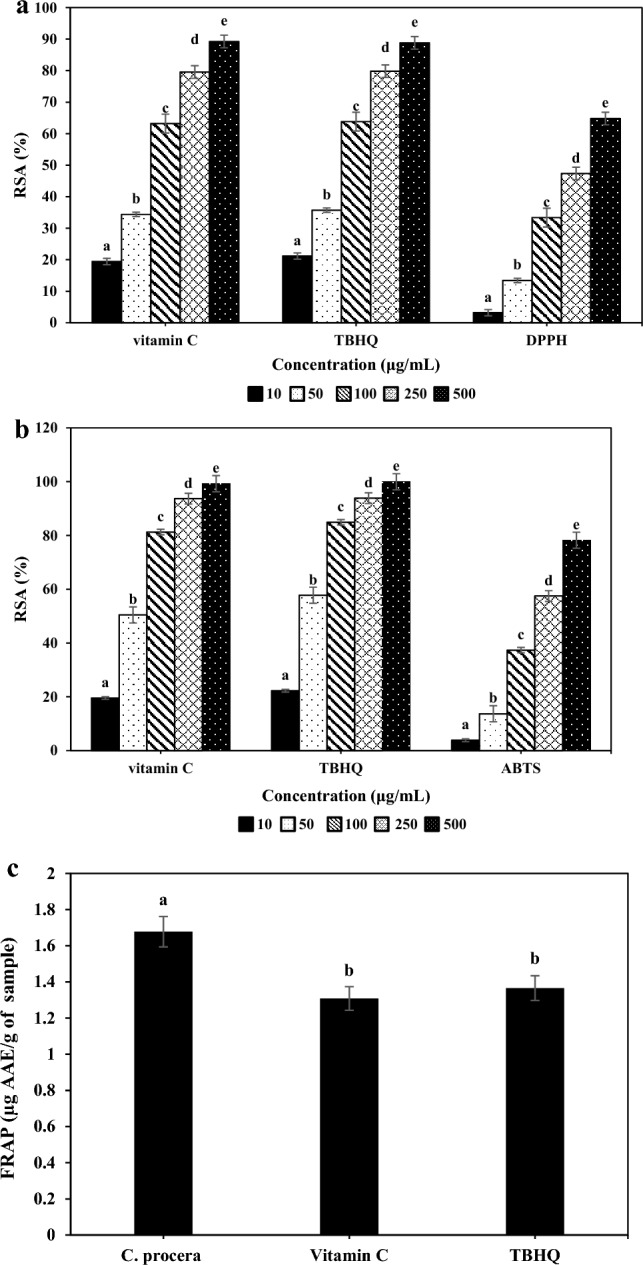
Determination of radical scavenging activity (RSA) percentage of *C. procera* extract on (**a**) DPPH, (**b**) ABTS, and **(c**) FRAP radicals. Letters a-e in the (**a** and **b**) indicate the difference between different concentrations in an antioxidant and in (**c**) **a**–**c** indicate difference between different antioxidants.

The antioxidant potential of *C. procera* extract was evaluated by examining its DPPH/ABTS radical scavenging activity and FRAP assay (Fig. 3). The graph related to the antioxidant activity of the extract, vitamin C (natural antioxidant), and TBHQ (synthetic antioxidant) in DPPH method is given in Fig. 3a. The IC_50_ values for extract, vitamin C, and TBHQ samples were 366.33 ± 10.5, 62.64 ± 3.5, and 59.67 ± 3.4 μg/mL, respectively. This indicates that the antioxidant activity of vitamin C and TBHQ is 5.8 and 6.13 times higher than that of the extract, respectively. Also, as the concentration of the extract increased, its antioxidant activity increased significantly (*p* < 0.05). There was no significant difference between the antioxidant activity of control samples, but they both differ significantly from the main sample (*p* < 0.05). The results related to the evaluation of antioxidant activity based on ABTS free radical scavenging are shown in Fig. 3b. As the concentration of the sample increased, its antioxidant activity also increased significantly, indicating a direct relationship between the concentration of the extract and its scavenging activity. Also, the antioxidant activity of vitamin C and TBHQ was significantly higher than that of the plant extract. The IC_50_ values for extract, vitamin C, and TBHQ samples were 169.04, 62.64, and 59.67 μg/mL, respectively. This indicates that the antioxidant activity of vitamin C and TBHQ was 2.70 and 2.83 times higher than that of the extract, respectively. The FRAP method was also used and its results are shown in Fig. 3c. The FRAP values for extract, vitamin C, and TBHQ samples were 1.67 ± 0.02, 1.3 ± 0.033, and 1.36 ± 0.026 µg AAE/g, respectively.

### Cytotoxic activity

**Figure 4:**
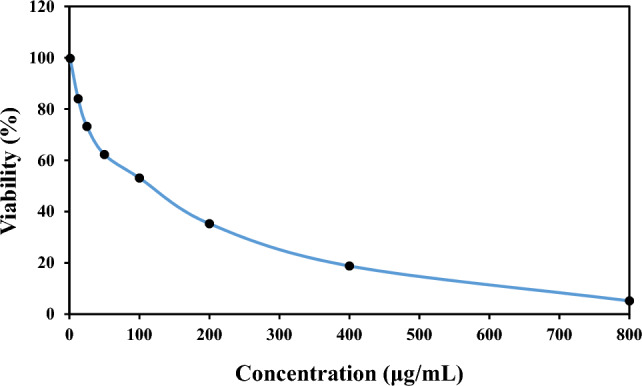
Cytotoxic effect of various concentrations of *C. procera* extract on survival of HT29 cell line.

Figure 4 shows the results related to the cytotoxicity of different concentrations of the extract. The survival rates of HT 29 cells in the presence of extract concentrations of 1, 12.5, 25, 50, 100, 200, 400, and 800 μg/mL extract were 99.76, 84.00, 73.19, 62.27, 53.11, 35.29, 18.81, and 5.19%, respectively. The IC_50_ value was calculated to be 236.87 ± 1.46 μg/mL. As shown in Fig. 4, the survival rate of cancer cells decreased as the concentration of the extract increased.

### Antibacterial activity

**Figure 5:**
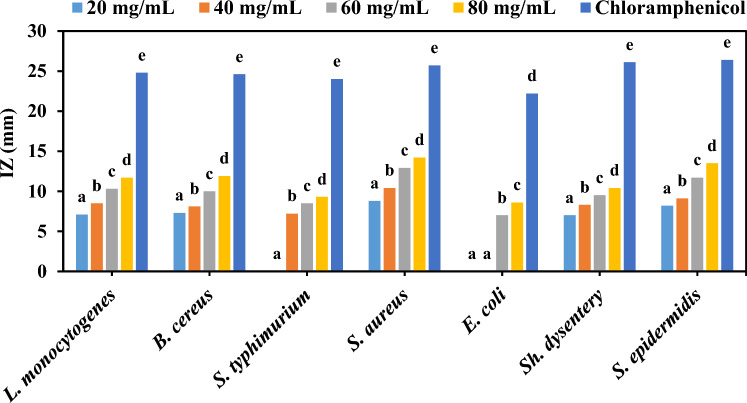
The average IZ (mm) of *C. procera* aqueous extract against pathogenic bacteria, based on DDA method (Different letters (**a**–e) in each strain show significant difference at *p* < 0.05).

The antimicrobial effect of the *C. procera* extract against the target bacteria was evaluated by the WDA, DDA, MIC, and MBC methods. Figure 5 shows the results of the DDA method. There was a significant difference between various concentrations of the extract (*p* < 0.05) and as the concentration of the extract increased, the IZ also increased. Moreover, the IZ of chloramphenicol antibiotic was significantly higher than that of the extract concentrations. The range of IZ created by the extract around the strains was between 0 (*E. coli* samples at concentrations of 20 and 40 mg/mL and *S. typhimurium* at 20 mg/mL) to 14.2 mm (*S. aureus* at a concentration of 80 mg/mL extract). *E. coli* (Gram-negative bacteria) and *S. aureus* (Gram-positive bacteria) strains had the highest and lowest resistance to the extract, respectively.

**Figure 6:**
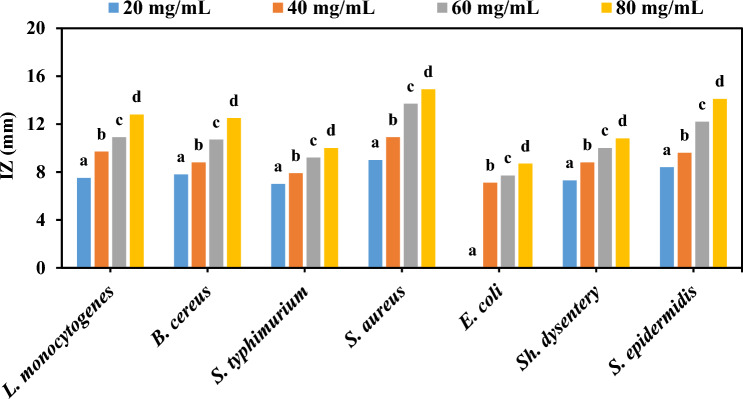
The average IZ (mm) of *C. procera* aqueous extract against pathogenic bacteria, based on WDA method (Different letters (**a**–**d**) in each strain show significant difference at *p* < 0.05).

In the WDA method, as shown in Fig. 6, the IZ created for *E. coli* was lower than for other strains. The lowest IZ for this strain was found in the presence of 20 mg/mL of the extract (*p* < 0.05). The highest IZ of 14.9 mm at a concentration of 80 mg/mL was established against *S. aureus*, resulting in an IZ range between 0 and 14.9 mm. As the concentration of the extract, the rate of inhibition against bacterial growth also increased significantly (*p* < 0.05). The results obtained from both methods are similar, indicating the effective antimicrobial activity of this extract.

Table 2 presents the MIC and MBC results of the *C. procera* extract. The results showed that *S. aureus* and *S. epidermidis* strains, with an MIC of 16 mg/mL, had the lowest resistance to the extract. On the other hand, *E. coli* and *S. typhimurium* strains, with an MIC of 128 mg/mL, had the highest resistance to the *C. procera* extract. The MBC value for *E. coli* (< 512 mg/mL) was higher than for other strains, while the lowest value was obtained for *S. aureus* and *S. epidermidis* (128 mg/mL).

**Table 2:** MIC and MBC of the aqueous extract of *C. procera* for some pathogenic bacteria.

Bacteria	MIC (mg/mL)	MBC (mg/mL)
*L. monocytogenes*	32	256
*B. cereus*	32	256
*S. typhimurium*	128	512
*S. aureus*	16	128
*E. coli*	128	> 512
*Sh. dysenteriae*	32	512
*S. epidermidis*	16	128

### Structural changes of extract-treated strains

**Figure 7:**
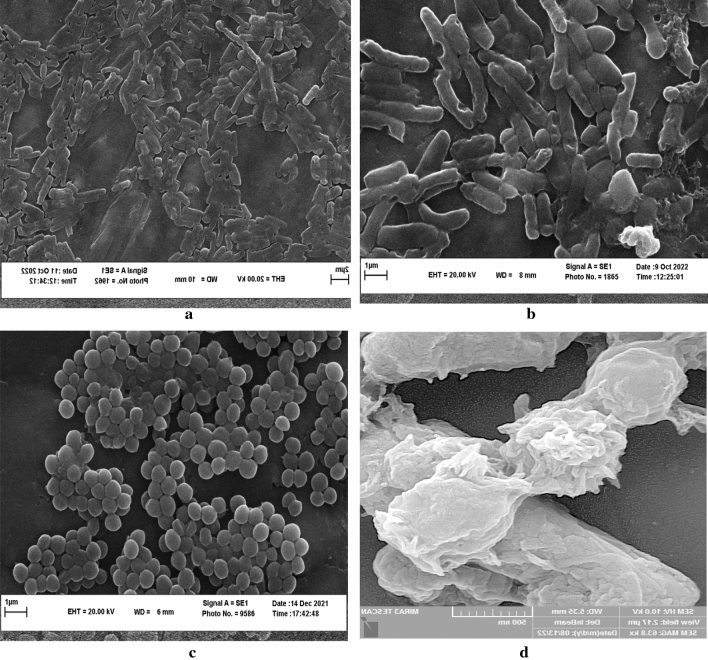
SEM images of *E. coli* (**a**), treated *E. coli* with *C. procer*a extract (**b**), *S. aureus* (**c**), and treated *S. aureus* with *C. procera* extract (**d**).

Antimicrobial compounds typically kill microorganisms by altering their structure and damaging their vital components. *E. coli* and *S. strains*, which had the highest and lowest resistance to the *C. procera* extract, respectively, were selected to evaluate the extract’s effect on their structure. The SEM images of *E. coli* showed that its normal rod structure is distorted (Fig. 7a). In the treated strain, the cell wall is damaged, torn, and perforated (Fig. 7b). Overall, it can be concluded that in *E. coli*, the extract increased cell permeability, destroyed membrane integrity, cut cell membrane, released cytoplasmic contents, and caused cell death. *S. aureus* is cocci-shaped and appears as clusters (Fig. 7c). In the image of *S. aureus* treated with extract (Fig. 7d), its cell structure was found to be changed and perforated, and its contents was released. The rupture of the cell wall can also be observed.

**Figure 8:**
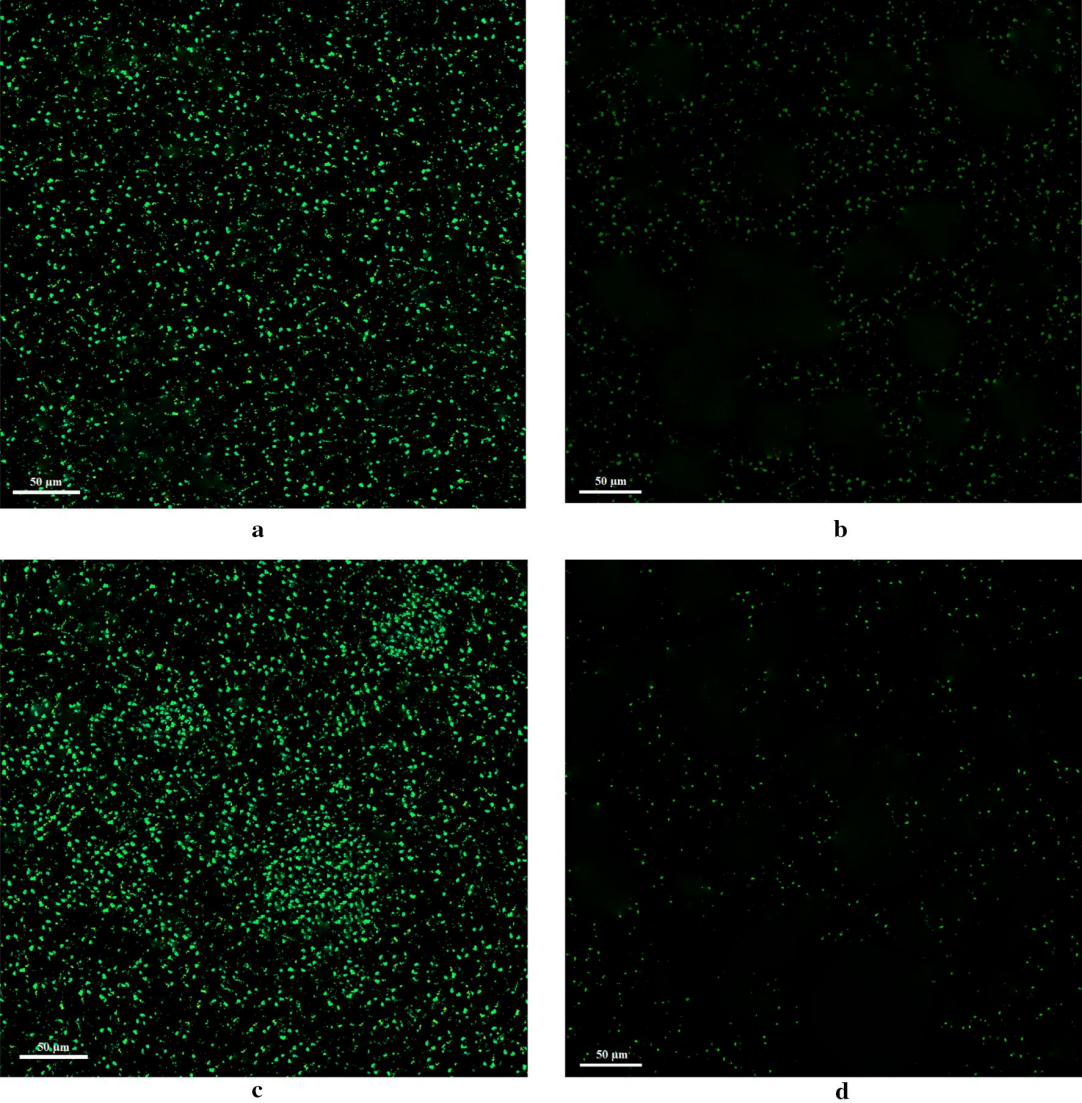
CLSM images of *E. coli* (**a**), treated *E. coli* with *C. procera* extract (**b**), *S. aureus* (**c**), and treated *S. aureus* with *C. procera* extract (**d**).

The CLSM was used to prepare images of control and treated *E. coli* and *S. aureus* strains. In the images obtained for both strains, a large number of green dots indicating living cells can be seen in the untreated samples (Fig. 8a,c). However, after treatment with the extract, the number of green dots (living cells) decreased significantly (Fig. 8b,d).

## Discussion

The *C. procera* extract was obtained using water extraction, as its main compounds, polyphenolic and flavonoid compounds, are polar and highly soluble in water. This is consistent with results reported in the literature^21,22^. Similarly, Song et al. (2019) and Sulaibi et al.^2,8^ reported that *p-*coumaric acid is one of the main constituents of *C. procera*. Another study detected kaempferol-3-O-rutinoside flavonoids (41.3 mg), isorhamnetin-3-O-rutinoside (27.4 mg), and quercetin-3-O-rutinoside (18.6 mg) in the *C. procera* extract, with kaempferol being identified as the main component of the extract^4^. The presence of rutin, kaempferol, and quercetin has also been reported in the organic extract of *C. procera*^23^. Previous studies have shown that flavonoids and polyphenols are the main constituents of *C. procera*^9^. A study of *C. procera* essential oil from Saudi Arabia and Egypt revealed the presence of 90 compounds, including mainly terpenes (sesquiterpenes and diterpenes), hydrocarbons, aromatics, and carotenoids^5^. This difference in compounds could be due to various factors such as climatic conditions, cultivation area and methods, plant species, and extraction methods. Also, the presence of the phenolic acids (caffeic acid and *p*-coumaric acid), and flavonoids (catechin, rutin, quercetin, and kaempferol) have been reported in *Piper betle L*., *Amaranthus gangeticus*, and *Lepidium draba* extracts^24–26^. According to the type of bonds identified in the FTIR spectrum of the sample and HPLC results, the presence of phenolic, carboxyl, benzene ring, propyl, aldehyde, cyclohexene, terpenoids (monoterpenes, sesquiterpenes and oxygenated derivatives), and phenylpropanoids was confirmed.

Phenolic compounds are known for their antioxidant properties; they can chelate redox-active metal ions and inhibit free radical chain reactions by preventing the conversion of hydroperoxides to reactive oxyradicals, which can then inactivate free radicals. Flavonoids are also known for their ability to scavenge free radicals by forming complexes with metal ions^27,28^. In line with our study, the leaf and skin solvent extracts of *C. procera* contained TPC of 20.41-100.18 mg GAE/g of DW and TFC of 18.33–92.92 mg catechin equivalent/g of DW^29^. The TPC and TFC of the *C. procera* extract were found to be in the moderate range when compared to extracts of some other plants, such as the methanol extract of *Ferulago angulata* (TPC of 72.33 mg GAE/g and TFC of 35.31 mg QE/g)^30^ and the hexane extract of *Thymus satureioides* (TPC of 97.9 mg GAE/g and TFC of 92.144 mg QE/g)^31^. Gholamshahi and Salehi Sardoei^32^ reported different TPC values for various parts of the plant, with the leaf having the highest TPC value (9.8 mg GAE/g of DW). Many factors can affect the amount of TPC and TFC, including extraction method, type of extracting solvent, weather conditions, cultivation method, type of plant, and different parts of the plant^33^. The presence of high amounts of biological molecules such as phenolic compounds and flavonoids has industrial and medicinal benefits, allowing for large-scale production of these plants and makeing agricultural business more competitive^24^. According to a study by Al-Rowaily et al. (2020), a high level of carotenoids was found in the *C. procera* extract^5^.

When oxygen is used to create cellular energy, a redox process occurs that releases free radicals, such as the hydroxyl radical, hydrogen peroxide, and super oxide anion^10^. The antioxidant properties of plant extracts were evaluated using various indices in several model systems to ensure their effectiveness. The *C. procera* extract obtained in this study demonstrated significant antioxidant activity. The IC_50_ value for leaf, fruit, flower, and latex *C. procera* extract based on DPPH radicals inhibition test was reported as 1.7, 0.21, 0.27 and 0.43 mg/mL, respectively^1^. Samani et al.^17^ achieved 63.69 and 64.33% free radical inhibition using DPPH and ABTS at a concentration of 1000 ppm of thyme essential oil. This difference in amount could be due to the extraction of the extract from different parts of the plant and the extraction method^1,34,35^. Secondary metabolites, particularly plant phenols and carotenoids, form a large group of compounds that act as primary antioxidants^11^. These agents have a high redox potential that enables them to act as scavengers, hydrogen donors, and single-electron oxygen scavengers. Studies have shown that excessive production of hydroxyl radicals, superoxide anions, and hydrogen peroxide can cause damage to protein oxidation and lipid peroxidation in living tissues and DNA cells^36^. In general, a plant’s antioxidant activity is determined by the amounts of its antioxidant compounds; plants with more phenolic compounds have higher antioxidant activity^37,38^. Additionally, antioxidant activity increases with increasing sample concentration, which is consistent with the findings of Ismail and Hong^39^. According to the results obtained from comparing the extract sample with vitamin C and TBHQ, the extract could be used as a substitute for synthetic antioxidants that have adverse effects on human health.

By the year 2030, it is predicted that there will be 26 million new cases of cancer and 17 million cancer-related deaths^10^. As a result, there is always a need for the development of novel anticancer drugs that are efficient and affordable. Considering the importance of medicinal plants in reducing cytotoxicity and their effect in decreasing carcinogenesis, the MTT method was used to measure the detoxification activity of the *C. procera* extract. Mathur et al. (2009) examined the cytotoxicity of methanol (CM), hexane (CH), water (CW), and ethyl acetate (CE) extracts of *C. procera* on Hep2 cells using the MTT method. They reported that the CE sample was the most potent growth inhibitor (96.3%) at 10 μg/mL, while the CM and CH samples had milder cytotoxic effect of 72.7 and 60.5%, respectively. The CW extract did not show any cytotoxic effect. The IC_50_ of CM, CH, and CE was calculated to be 10 µg/mL^40^. Oliveira et al. (2007) studied the cytotoxic activity of laticifer proteins (LP) recovered from the latex of *C. procera* and found significant cytotoxicity with IC_50_ values ranging from 0.42 to 1.36 µg/mL against SF295 and MDA-cell lines^41^. Since there is a direct relationship between antioxidant and anticancer activity, the cytotoxic effect is increased as the levels of antioxidant compounds such as polyphenols, carotenoids, and tannins are increased. Antioxidant compounds play an important role in human health by inhibiting free radicals and chelating toxic compounds^14^.

Even though the pharmaceutical industry is consistently producing new antibiotics, the number of microorganisms that are resistant to chemical antimicrobial drugs poses a serious threat to the management of infectious diseases^42,43^. As a result, new, highly resistant bacterial strains appear, which is extremely dangerous, especially for people with weakened immune systems. In order to create bioactive antimicrobial agents with low toxicity, a wide spectrum, and good pharmacokinetics that can be used in clinical settings without requiring any chemical modification, natural plant products serve as a constant source of inspiration^10,44–48^. Recently, there has been a push to promote the use of plants as complementary medicines for treating infectious diseases. These plants can serve as suitable substitute for preservatives in the industry, reducing both their toxic effects on the body and production costs. Nenaah et al. (2013) reported that the IZ created by *C. procera* extract against bacteria ranged from 8.5 to 28.5 and from 10.5 to 30 mm against fungal strains. They found that the methanolic extract of *C. procera* had better antimicrobial activity than other samples, with an IZ ranging from 9.5 to 22.5 mm, while ether and chloroform petroleum extracts showed no antibacterial activity in some cases^4^. Yesmin et al. (2008) reported that the crude methanolic extract of *C. procera* at a concentration of 500 µg/mL produced moderate antibacterial activity against *S. aureus* and *S. epidermidis* using the WDA, with the IZ values ranging from 6 to 22 mm^49^. Similarly, Kareem et al. (2018) reported that ethanol extracts of *C. procera* leaves and latex had moderate antimicrobial effects against *E. coli* bacteria, with an IZ of 14.1 mm^50^. Neenah and Ahmad (2011) investigated the antimicrobial activity of *C. procera* extract and reported MIC and MBC values of 0.25 mg/mL and 0.75 mg/mL against the investigated microorganisms, respectively. The MIC of almost all plant extracts against pathogenic bacteria has been reported to range from 0.04 to 0.32 mg/mL^51^. Rigano et al. (2009) reported significant antimicrobial activity for flavonoids such as quercetin and kaempferol^52^. Antimicrobial activity of the extract against *E. coli* and *P. aeruginosa* (MIC 90 µg/mL) and *Aspergillus fumigatus* (MIC 130 µg/mL) has also been reported^41^. Additionally, Saddiq et al.^53^ examined the antimicrobial activity of *C. procera* ethanolic extract using the WDA assay and MIC against six pathogenic microbial strains (*Candida albicans*, *Aspergillus fumigatus*, *S. aureus*, *Bacillus subtilis*, *E. coli*, and *Klebsiella pneumonia*). The extract was found to be significantly activity against *S. aureus*, *K. pneumonia*, and *E. coli*, with inhibition zones of 18.66 mm, 21.26 mm, and 21.93 mm, respectively. The MIC of the plant extract was found to range from 0.60–1.50 mg/mL, indicating that it is a moderate inhibitor of *B. subtilis*^53^.

The antimicrobial activity of a plant extract is generally attributed to the chemical compounds present in the plant mixture. The maximum effectiveness of a medicinal plant may result from the interaction of several different constituents rather than from the presence of just one main active ingredient^34,50^. The main reason for the differences in antimicrobial activity is the varying amounts of secondary metabolites in the plant, which can be influenced by factors such as species and extraction methods^14^. Gram-positive bacteria have a single cell wall structure, while Gram-negative bacteria have a multi-layered cell wall structure, making them more resistant to antimicrobial compounds^54,55^. The antimicrobial activity of plant flavonoids against different microbial species has become increasingly important in recent years. The antimicrobial effect of plant extracts is generally due to phenolic compounds with free hydroxyl groups, such as flavonoid compounds, which can act in several ways: forming bonds with extracellular and soluble proteins, glutamate and phosphate of bacteria; altering cellular peptidoglycan; disrupting bacterial membrane permeability; inhibiting vital enzyme pathways; binding to the active site of enzymes; forming hydrogen bonds with enzymes; and altering enzyme metabolism^4,56^.

The extract caused significant changes in the structure of *E. coli* and *S. aureus*. Similar observations were made by Moghayedi et al. (2017), who found that treated *E. coli* had an incomplete and deformed shape with an absent cell wall under the effect of extract^57^. Alizadeh Behbahani and colleagues (2020) demonstrated that the essential oil of *Cinnamomum zeylanicum* could damage *E. coli* cell membranes and facilitate intracellular compound leakage, as evidenced by the presence of deformed and sunken cell shapes in the SEM images^18^. Shikonin treatment of *L. monocytogenes* resulted in reduced biofilm adhesion, altered biofilm morphology, and disruption of biofilm architecture, as verified by light microscopy and field-emission scanning electron microscopy^58^. In general, the antimicrobial compounds of the extract (polyphenols, tannins, and carotenoids) altered the natural structure of bacteria, leading to dysfunction and ultimately cell death.

## Conclusion

The study aimed to investigate the potential of *C. procera* leaf aqueous extract in terms of its phytochemical compounds, antioxidant, anticancer, and antimicrobial properties. According to the results obtained from HPLC and FTIR, the main compounds of *C. procera* extract were catechin, rutin, *p*-coumaric acid, caffeic acid, luteolin, and kaempferol. The extract has good antioxidant effect and high antimicrobial power, especially against Gram-positive bacteria. The most resistant strain was *E. coli* and the most sensitive was *S. aureus*. The morphology of these bacteria was affected by the extract, as shown by examination through SEM and CLSM. In general, this study showed that the *C. procera* plant has the potential to be used in the food and medicine industry due to its antioxidant, anticancer, and antimicrobial properties. However, the aqueous extract of *C. procera* should be subjected to bio-guided fractionation in order to isolate the active ingredients responsible for the corresponding biological activity.

## Data Availability

All data relevant to the study are included in the article.
